# Commentary: Higher blood manganese level associated with increased risk of adult latent tuberculosis infection in the US population

**DOI:** 10.3389/fpubh.2025.1705799

**Published:** 2025-11-26

**Authors:** Yu Dai, Xiaobao Chen, Cheng Wang, Panfeng Shang

**Affiliations:** 1 Department of Urology, Baoji People's Hospital, Baoji, Shaanxi, China; 2 Department of Urology, Lanzhou University Second Hospital, Lanzhou, Gansu, China

**Keywords:** blood manganese, latent tuberculosis infection (LTBI), NHANES, selection bias, data transparency

## Introduction

We read with great interest the article by Qi et al. (1) titled “Higher Blood Manganese Level Is Associated with Increased Risk of Adult Latent Tuberculosis Infection,” published in Frontiers in Public Health. Which explores the novel association between blood manganese and latent tuberculosis infection using the NHANES database. The study addresses an important public health question, and we commend the authors for their comprehensive analyses, including multivariate adjustment and restricted cubic splines.

However, we have identified a critical and mathematically implausible discrepancy in the reported participant flow, as detailed in Figure 1. The authors state that from the 5,158 adults with TB infection status, 3,448 were excluded due to “missing data of blood heavy metals.” This claim is fundamentally irreconcilable with the public NHANES data. The 2011–2012 laboratory files document that complete data for all five blood heavy metals (Pb, Cd, Hg, Se, and Mn) are available for 7,920 participants. Given that the authors' starting point for this exclusion is a sub-cohort of 5,158 individuals, it is numerically impossible for 3,448 of them (67% of the sub-cohort) to be missing the very data that exists for 7,920 individuals in the overall dataset. This profound inconsistency indicates that the reported exclusion criterion is inaccurate and that the actual process for selecting the final 1,710 participants involved undisclosed filters, raising grave concerns about selection bias.

Multiple studies using the same NHANES 2011–2012 cycle to analyze blood metals report final sample sizes and exclusion numbers that are consistent with the public data and vastly different from Qi et al.: Wu et al. (2) (Frontiers in Nutrition, 2023) retained 7,108 participants: Chen et al. (3) (Journal of Affective Disorders 2024) retained 4,000 participants: Zhou et al. (4) (Translational Medicine Journal, 2025) retained 3,576 adults after excluding only 172 for missing heavy metals data.

The exclusion of 3,448 individuals by Qi et al. (1) is a profound outlier, indicating the application of unique and unreported select: The combination of an implausible exclusion reason, a high risk of selection bias, and the lack of sensitivity analysis fundamentally undermines the scientific validity of the study. The reported association may not be generalizable and could be severely biased; to address these concerns and ensure the integrity of the published record, we respectfully request that the authors; publicly clarify the specific, individual-level reasons for excluding 3,448 participants. Provide a comparison of baseline characteristics between the included and excluded cohorts to assess the potential magnitude of selection bias. Perform and report a sensitivity analysis using a consensus-based, reproducible participant flow (e.g., starting from all adults with defined LTBI status and excluding only those with missing manganese data) and present the resulting effect estimate. Until a satisfactory explanation and supporting analyses are provided, we urge the readership to interpret the reported associations with extreme caution.

## Discussion

Transparency in participant selection is paramount in observational research, as it is foundational to assessing bias and ensuring reproducibility (5). The internal inconsistency between the stated exclusion reason and the presented data, coupled with the extreme scale of exclusion compared to peer studies (2, 4), prevents a clear understanding of how the final study sample was constituted. We urge readers to interpret the reported findings with caution. We also respectfully call upon the authors to clarify the specific variables that were missing for the excluded individuals and to provide a detailed account of all applied selection criteria to ensure the reproducibility of their work.
